# Cocaine as a Local Anæsthetic

**Published:** 1887-08

**Authors:** 


### Cocaine as a Local Anæsthetic.

Knowing that cocaine is not well absorbed by the skin, and therefore does not produce much of an anæsthetic effect, Dr. Wagner, of Vienna, was led to make experiments for the purpose of ascertaining a method by which a better absorption and anæsthesia might be produced. Basing his theory upon the established principle that fluids move from the positive to the negative pole in a galvanic current, he saturated the positive electrode with a cocaine solution, applied it to the skin, and applied the negative pole a short distance from the positive. As the result seemed satisfactory, he made a number similar experiments in the clinic of Billroth to determine the value of the method in surgical practice. He found that by keeping the positive electrode saturated with a sufficiently strong solution, and allowing the current to pass in the manner described for a short time, incisions could be made in the skin without producing any pain. His results and the description of the method were presented to the Society of Physicians of Vienna at their meeting February 5, 1886.

In regard to this method, I may say that I have personally made a number of experiments with a view of ascertaining its merits, which I intend to publish soon in the form of a paper, but which would occupy too much time were I to report them here. I will merely say that in my hands the matter is a success, and in my opinion one of the most useful discoveries in dermatology which have been made during the year 1886. In my experience, in the operation for the removal of superfluous hairs by electrolysis, and in other minor operations of the skin, the patient need experience absolutely no pain whatever.

